# Design, Optimization and Improvement of FBG Flexible Sensor for Slope Displacement Profiles Measurement

**DOI:** 10.3390/s19173750

**Published:** 2019-08-30

**Authors:** Changbin Tian, Zhengfang Wang, Qingmei Sui, Jing Wang, Yanan Dong

**Affiliations:** School of Control Science and Engineering, Shandong University, Jinan 250061, China

**Keywords:** FBG, displacement profiles measurement, segmental correction method, strain increments, clustering algorithm

## Abstract

The accurate measurement of slope displacement profiles using a fiber Bragg grating flexible sensor is limited due to the influence of accumulative measurement errors. The measurement errors vary with the deformation forms of the sensor, which dramatically affects the measurement accuracy of the slope displacement profiles. To tackle the limitations and improve the measurement precision of displacement profiles, a segmental correction method based on strain increments clustering was proposed. A K-means clustering algorithm was used to automatically identify the deformation segments of a flexible sensor with different bending shapes. Then, the particle swarm optimization method was adopted to determine the correction coefficients corresponding to different deformation segments. Both finite element simulations and experiments were performed to validate the superiority of the proposed method. The experimental results indicated that the mean absolute errors (MAEs) percentages of the reconstructed displacements using the proposed method for six different bending shapes were 1.87%, 5.28%, 6.98%, 7.62%, 4.16% and 8.31%, respectively, which had improved the accuracy by 26.83%, 18.94%, 29.49%, 26.35%, 7.39%, and 19.65%, respectively. Therefore, it was confirmed that the proposed correction method was competent for effectively mitigating the measurement errors and improving the measurement accuracy of slope displacement profiles, and it presented a vital significance and application promotion value.

## 1. Introduction

Landslide is part of the most disastrous geological hazards, occurring due to rainfall, earthquake load, steep slopes and human activities, and causing thousands of casualties, significant damage, and economic loss [1,2]. In the Shuping landslide, in May 2004, approximately 163 families (around 580 people) were relocated. Recently, at least 10 people were killed and 22 others are missing after a rain-triggered landslide buried mountain-side factories in northwestern Shaanxi province. Internal horizontal displacements of slope can provide deformations at different depths and determine the magnitude, rate, direction, depth, and type of landslide movement. Thus, it is important for researchers to understand the deformation mechanisms of landslides, analyze the slope stability, provide an early warning to the public, and protect lives and property [3,4].

In the past several decades, various geotechnical instruments and measurement methods have been developed and adopted for measuring the internal displacements of slopes [5]. Traditional measurement methods mainly consist of an inclinometer [6], linear variable differential transformers (LVDTs) [7], an inductive displacement sensor [8], and multi-point extensometers [9]. A corresponding evaluation with respect to the slope stability can be implemented by analyzing displacement distributions measured using the above sensors. In spite of this, it shows several disadvantages, including poor moisture-proofness, signal loss for long distance transmission, poor durability, and the fact that active devices require power supply, etc. As a particular class of sensors, fiber Bragg grating (FBG) sensors have received much attention due to their prominent advantages, such as being immune to electromagnetic noise, being waterproof, easy multiplexing, a negligible weight and size, high precision, etc. [10,11]. A variety of FBG-based sensors [12], such as the FBG-segmented deflectometer, FBG strain gauge and FBG tilt sensor, have been designed for the real-time measurement of different mechanical parameters, including internal displacements, strains and inclination angles, etc., so as to conduct a safety evaluation of slopes. Nevertheless, most of the aforementioned FBG sensors can only monitor the parameters that are related to slope stability with the point-mode. They are inapplicable for measuring the whole internal displacement profiles of the slope and for locating the sliding surfaces.

To tackle the above-mentioned limitations, some FBG-embedded flexible sensors that were fabricated by integrating the FBG arrays into flexible substrates have been widely adopted to capture the displacement profiles in the field of robotics, medical engineering, aerospace, infrastructure and geotechnical engineering [13,14]. These FBG flexible sensors could be pre-installed in the structure to be measured, utilizing shape reconstruction algorithms to reconstruct the displacement profile distributions. The deformed shape reconstructions of flexible sensors are crucial to accurately measure the structural displacement profiles. Bhamber et al. [15] and Xu et al. [16] adopted bidirectional curvatures and a curve fitting algorithm to reconstruct the shapes of the robotic arm and soft surgical actuator. Yi et al. [17] developed a shape reconstruction method based on spatial movable coordinates, and realized the reconstruction of the flexible morphing wing shape. Derkevorkian et al. [18] utilized strain information of the swept plate structure and a series of displacement transfer functions to estimate the corresponding displacement fields. Kim et al. [19] investigated a deflection estimation technique for measuring the shape of rotating blades, which was based on distributed strain information and a displacement-strain transformation (DST) matrix obtained from the modal approach. Bang et al. [20] created a finite element model based DST matrix for the estimation of wind turbine structures. In the field of geotechnical engineering, researchers have studied shape sensing methods for measuring displacement profiles in critical areas based on the FBG-embedded flexible sensors. Wang et al. [21] fabricated an intelligent geogrid embedded with FBG sensors and employed a curvature-based reconstruction method to estimate the displacements related to geotechnical engineering. Kim et al. [22] utilized a regression analysis method to measure the deflection curves, and subsequently adopted the method for the evaluation of bridge displacements. Li et al. [23] and Zhu et al. [24] established strain-deflection relationships for FBG flexible rods, which were then used in physical model tests to measure displacement profiles in an underground cavern group and dam. As far as monitoring the internal displacements of slopes was concerned, Pei et al. [25] developed a new type of FBG-based in-place inclinometer for slope monitoring according to the classical indeterminate beam theory. Guo et al. [26] estimated the displacement profiles of the slope using the curvatures and deflection angles detected by the FBG flexible sensor. The above scholars, with comprehensive insights in the field of FBG shapes sensing, have propelled research forward, and the technology has advanced to achieve an excellent performance in their respective application fields.

In practical applications, encounters with accumulative measurement errors are unavoidable during the displacement profile reconstruction employing that abovementioned methods. This is due to the temperature impacting the FBG points, the differences in the strain transfer rates, and the layout intervals of FBG sensing points, etc. These inefficiencies can be overcome by using the suitable correction method for correcting the reconstructed displacements, as well as by improving the measurement accuracy. So far, some works have already made progress in the error analyses and corrections of shape sensing. Sun et al. [27] experimentally calibrated the relationships between bending curvatures and wavelength-shifts at each sensing point in order to reduce strain transferring errors, and adopted a linear interpolation of the curvatures to improve the reconstruction accuracy of polyimide film deformations. Abayazid et al. [28,29,30] optimized the placement of sensing points and used the k-nearest neighbor interpolation model to reconstruct the curvature functions, and the proposed methods were conducted to improve the reconstruction precision of structural deformations. Zhang et al. [31] developed the least mean square algorithm based parameter identification method and the construction of the dynamic error analysis model, which has been employed to shape reconstructions and error analyses of space plate structures. Wang et al. [32] investigated an in-situ calibrated deformation reconstruction method, and improved the estimation accuracy of deformation fields effectively.

Although the correction methods being leveraged in the aforementioned studies have mitigated the accumulative errors and improved the accuracy of measured displacement profiles, they corrected the measured displacements without considering the diversity of deformation forms. It was found in the author’s previous research [33] that the correction method depended highly on the deformation forms. The displacements with a variety of deformed configurations cannot be universally corrected using one established and unique correction coefficient. The best way is to determine the correction weights for different bending shapes and then correct the reconstructed displacements accordingly. For the task of the internal horizontal displacements monitoring in the slopes, the displacements to be measured sometimes cover a long-distance range in the vertical direction. Furthermore, the displacements at different depths of the slopes are extremely complex during the evolution process of landslides. As a result, the internal horizontal displacements may have a variety of deformed shapes along dissimilar positions of the slope. Therefore, the displacement profile sensing method and correction method for monitoring the internal displacements should be able to automatically identify the deformation segments with different bending shapes, and then correct the different deformation segments according to the corresponding correction coefficients. Nevertheless, the methods of automatic identification of deformation segments and segmental correction have seldom been studied. Therefore, the ultimate goal of the current research is to develop a displacement profile reconstruction and correction method dedicated to the FBG flexible sensor for the internal displacement profiles monitoring of the slope.

In this paper, we proposed a segmental correction method based on strain increment clustering with the characteristics of self-correction displacement profile reconstruction, which had the ability to identify and correct deformation segments automatically for different bending shapes of the flexible sensor. Specifically, we calculated the strain increments at sensing points and extracted them as eigenvalues to be clustered. Then the clustering algorithm was employed to cluster the strain increments so that the deformation segments with different bending shapes of the flexible sensor could be identified automatically. The correction coefficients of the deformation segments were determined by calibrating the typical bending shapes of the sensor in the measurement of the slope displacement profiles, and the optimization algorithm was adopted to determine their optimal values. The remainder of the paper was organized as follows: in Section 2, the correction method of shape sensing was explained in detail. Then, in Section 3, a finite element simulation was conducted to verify the displacements sensing effects. Next, we conducted an FBG flexible sensor fabrication, experiment and analysis in Section 4. Finally, Section 5 presents the concluding remarks.

## 2. Methodology

### 2.1. Reconstruction Based on Beam Element Decomposition Method

The flexible sensors for the slope displacement monitoring are mostly manufactured by flexible structures embedded within FBG. FBG sensing points are deployed at equal intervals along the axis of the flexible structures. It should be emphasized that the deformation displacements of the slope are calculated according to the strain distributions measured by the quasi-distributed FBG sensing points embedded in the flexible substrates. The flexible structures can be considered to be composed of several beams with a length *L* and the end of each beam being called the measuring point.

**Figure 1:**
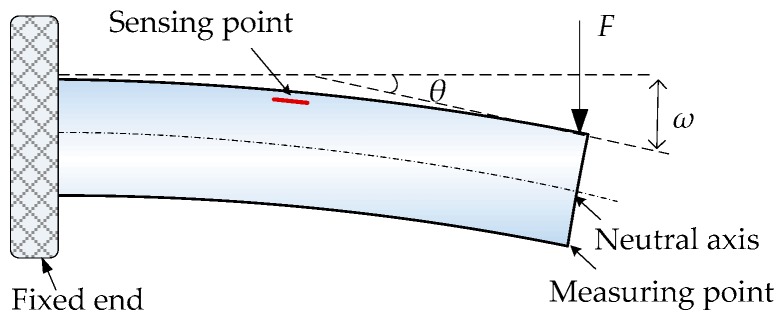
Measurement mechanism of each of the beam.

As described in Figure 1, the beam is bent under the action of force *F* at the measuring point, which can be modeled by the pure bending model in mechanical engineering [34,35]. The distance from the sensing point to the fixed end is *L*/2. According to material mechanics, the strain of the sensing point can be expressed by:

(1) $$\Delta \varepsilon =\frac{FLZ}{2EI}$$

where *E* is the elasticity modulus, *I* is the inertia moment, and *Z* is the distance between the sensing point and the neutral axis.

Therefore, the deflection *ω* and rotation angle *θ* at the measuring point can be represented as:

(2) $$\left\{ \begin{array}{l} \omega =\frac{FL^{3}}{3EI}=\frac{2L^{2}}{3Z}\Delta \varepsilon \\ \theta =\frac{FL^{2}}{2EI}=\frac{L}{Z}\Delta \varepsilon \end{array}\right.$$

**Figure 2:**
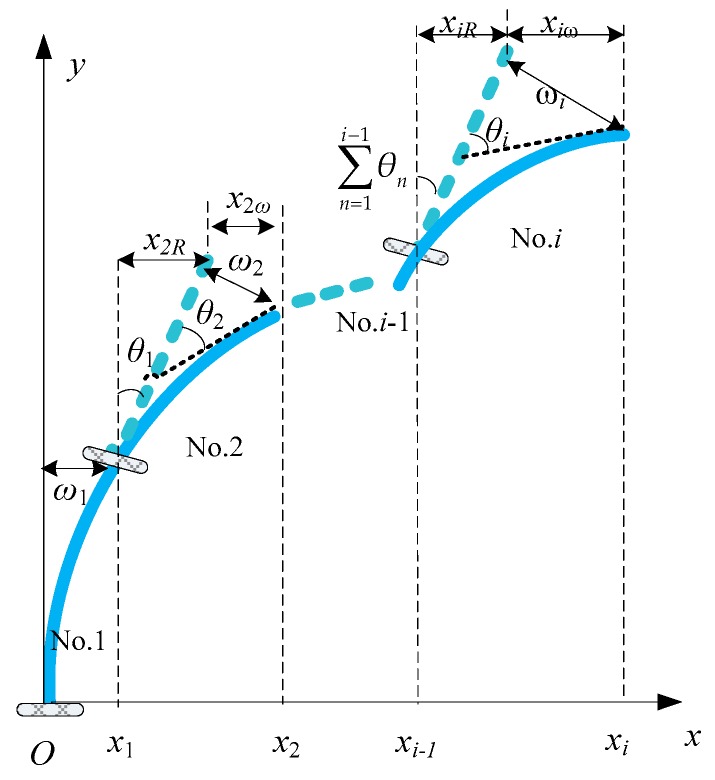
The schematic diagram of the beam element decomposition method.

In a geometric coordinate system, the fixed end coordinate of the first beam is *O* (0, 0) and the tangent direction is *y*-axis, as shown in Figure 2.

The horizontal displacement of the first beam at the measuring point is:

(3) $$x_{1}=\omega_{1}$$

When *i* ≥ 2, for the *i*-th beam, the horizontal displacement of the measuring point consists of three parts: the measuring point displacement of the (*i*−1)-th beam *x_i_*_−1_, the sum of the rotation angles of the (*i*−1)-section beams induce the displacement *x_iR_*, and *x_iω_* is produced by the deflection *ω_i_* of the *i*-th beam. *x_iR_* and *x_iω_* are represented as:

(4) $$\left\{ \begin{array}{l} x_{iR}=L\cdot \sin\sum_{n=1}^{i-1}\theta_{n}\\ x_{i\omega }=\omega_{i}\cdot \cos\sum_{n=1}^{i-1}\theta_{n} \end{array}\right.$$

According to the above description, we can obtain the following formulas:

(5) $$\left\{ \begin{array}{ll} x_{1}=\omega_{1} & i=1\\ x_{i}=x_{i-1}+L\cdot \sin\sum_{n=1}^{i-1}\theta_{n}+\omega_{i}\cdot \cos\sum_{n=1}^{i-1}\theta_{n} & i>1 \end{array}\right.$$

Therefore, the displacements of the measuring points will be deduced from the strain values measured by the FBG sensing points. It should be emphasized that the rigidity of the flexible rod is consistent with or less than that of the whole soil environment medium in the slope where the flexible rod is located. Thus, it can be considered that the deformation of the flexible rod reflects the displacements distributions at different depths of the slope.

### 2.2. Correction Method

The shape reconstruction algorithm based on a beam element decomposition method has the ability to calculate the bending shapes of the FBG flexible sensor. However, the occurrence of accumulative errors during the deformed shapes reconstruction is inevitable, and the larger the measurement range, the more serious the accumulative errors are. Additionally, the displacements to be measured sometimes cover a long-distance range in the vertical direction for the internal horizontal displacements monitoring in the slopes. Furthermore, the displacements at different depths of the slopes are extremely complex during the evolution process of landslides. As a result, the internal horizontal displacements may have a variety of deformed shapes along dissimilar positions of the slope. Therefore, the correction utilizing unified coefficient can hardly guarantee the measurement accuracy for the diversity of deformation forms.

To tackle this problem, a segmental correction method based on strain increment clustering with the characteristics of self-correction displacement profiles reconstruction was proposed, which had ability to automatically identify different deformation segments and correct the reconstructed displacements of each segment accordingly. Specifically, we calculated the strain increments at sensing points and extracted them as eigenvalues to be clustered. Then, the clustering algorithm was employed to cluster the strain increments so that the deformation segments with different bending shapes of the flexible sensor could be identified automatically. The correction coefficients of the deformation segments were determined through calibrating the typical bending shapes of the sensor in the measurement of the slope displacement profiles, and the optimization algorithm was adopted to determine their optimal values. The specific scheme is as follows.

#### 2.2.1. Categories of Internal Displacements

**Figure 3:**
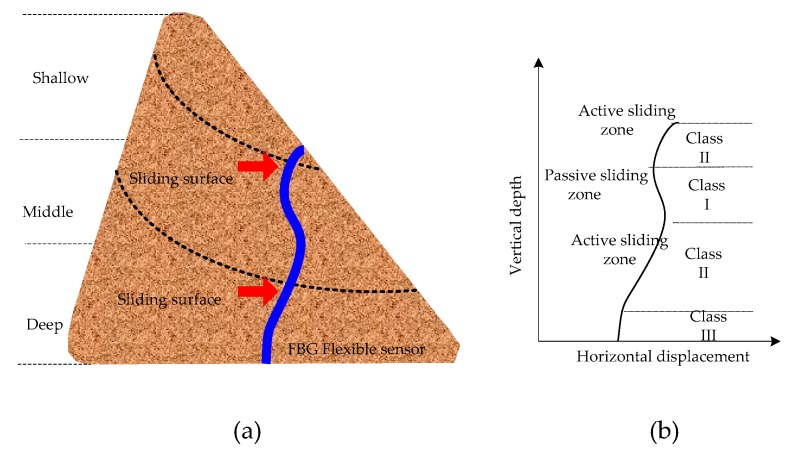
(**a**) Schematic diagram of the internal displacements of the slope monitored by the flexible sensor; (**b**) Internal displacement profiles of the slope.

The internal horizontal displacements of the slope can provide information on the below-ground movement and can serve to detect the position of the sliding surfaces, which are crucial to understand the possibility of the occurrence and mechanism of landslide events. It is necessary to lay flexible sensors in the places where deformation may occur according to the actual geographical location to effectively monitor the internal displacement of the slope. In the process of FBG flexible sensor placement, firstly, drilling is carried out in the slope, and the casing with the same material of flexible substrate is embedded in the borehole. Then, the outer periphery of the casing is grouted with cement mortar. Next, the FBG flexible sensor is placed in the casing [36,37]. With the protection of the casing, even if the shear strains around the FBG flexible sensor are large, this will not cause the plastic deformation of the whole sensor structure. Meanwhile, it avoids direct contact with the backfill, which may cause potential damage to the FBG sensing points. The casing and flexible substrate are the same material, which can achieve a better mechanical matching. The FBG flexible sensor is installed vertically in a slope borehole and traverses the potential sliding zones to the bottom (see Figure 3a). The bottom of the flexible sensor is considered fixed, and the deformation sensing mechanism is similar to that of a cantilever beam. The shear force of the internal soil is altered as a result of rainfall, groundwater, earthquake load, overload and other factors, which eventually trigger the landslide. According to the thickness of the landslide, it can be divided into a shallow landslide, middle landslide and deep landslide. The deformed shapes of the flexible sensor have diversified characteristics because multiple sliding surfaces may occur at different depths of the slope, and the displacements of the active sliding zone and passive sliding zone are different. As shown in Figure 3b, the bottom displacements of the slope are basically unchanged, and the displacements in the active sliding zones are larger, while the displacements in the passive sliding zone are smaller. It is found that although the flexible sensor has many different bending shapes, they can be categorized into three classes based on the strain increments at the sensing points. In Class I, the strain increments at the sensing points are positive, while the negative strain increments at the sensing points are Class II, and the strain increments hardly changed in Class III. The displacements of the measuring points are calculated based on the strain values measured by the FBG sensing points. If the strain value of the sensing point remains untouched, the beam where the sensing point is located will not produce a deflection displacement. Thus, only the strain values of the sensing points at Classes I and II are corrected, but the strain values of Class III are not corrected. A clustering method based on strain increments has been proposed according to the above analyses.

#### 2.2.2. A Clustering Method Based on Strain Increments

The strain increments at the sensing points are clustered, achieving the goal of segmental correction for different bending shapes of the flexible sensor. We assume that we start from the fixed end of the flexible sensor and the definition of the strain increment as the strain variation of the *i*-th sensing point relative to the (*i*−1)-th sensing point. Specifically, central wavelengths of the FBG sensing points are collected using an FBG demodulator, and Equation (9) is applied to convert the wavelength variations to the strain values S*_i_*. Then, we calculate the strain increment A*_i_* at each FBG sensing point. When *i* > 1, A*_i_* = S*_i_* − S*_i_*_−1_; when *i* = 1, A_1_ = A_2_. We determine whether |A*_i_*| is greater than 10^−5^, and if so, A*_i_* remains unchanged; if not, make A*_i_* = 10^−6^. The demodulation precision of the FBG interrogator is 1 pm, which is equivalent to about 1 micro-strain. The absolute values of all strain increments under 10 micro-strains are regarded as constant to increase fault tolerance. For further convenience of calculation, the points with constant absolute values of strain increments are assigned as 10^−6^. The strain increments at the sensing points are clustered directly, and will result in clustering errors because of the inflection points of the strain values.

To eliminate clustering errors caused by inflection points, the strain incremental ratio B*_i_* at each FBG sensing point is calculated. When *i* > 1, B*_i_* = A*_i_*/A*_i_*_−1_; when *i* = 1, make B_1_ = B_2_. Then, we determine the number of |B*_i_*| > 10 or |B*_i_*| < 0.1, which is equal to the number of inflection points. The strain increments of the same categories change slightly, so the strain incremental ratios are on the brink of 1. The inflection points change the variation trend of the strains at the sensing points. The strain value at the inflection point is the maximum value, where the absolute value of the strain incremental ratio is less than 0.1. While the strain value at the inflection point is the minimum value, the absolute value of the strain incremental ratio at this point is greater than 10. The strain increment A*_i_* at the inflection point is replaced by A*_i_*_+1_. The new obtained strain increments are clustered based on an intelligent clustering algorithm, and the bending shapes of the flexible sensor are divided into different deformation segments. The strain values of the different deformation segments are corrected by corresponding correction coefficients. The bending shapes of the flexible sensor are reconstructed by the beam element decomposition method and the corrected strains.

#### 2.2.3. Cluster Algorithm and Optimization Algorithm

The K-means clustering algorithm is adopted to intelligently cluster deformation segments under different bending shapes of the flexible sensors in this research study. K-means clustering is a classical clustering algorithm and the widely applicable one, developed by Mac Queen in 1967 [38]. This method is a local clustering algorithm that divides the whole dataset into K disjoint clusters. We determine whether there are points where |A*_i_*| are less than 10^−5^, and if not, we make K = 2; if so, we determine whether the number of inflection points is greater than 1, and if so, we make K = 3; if not, we make K = 2. We distribute each data point to the nearest cluster based on the Euclidean distance matrix. The clustering results obtained by this algorithm are marked by small sample distances within the clusters and large sample distances between the clusters. Compared with conventional popular clustering algorithms, this algorithm has the characteristics of a fast convergence to a local optimum and sizable amount of datasets processing. The different deformation segments of the flexible sensor are clustered automatically based on the K-means algorithm, and the corresponding correction coefficients are automatically selected for the different segments. The process of calculating the correction coefficients is described as follows.

We assume that the actual horizontal displacements of the measuring points are *X_Ri_*, with *I* = 1, 2, …, *n*. The calculation displacements based on the corrected strain values *k*·Δ*ε* and the reconstruction algorithm are *X_Ci_* (*k*). Thus, the mean absolute errors (MAEs) of the measuring points are calculated in accordance with the following formula:

(6) $$\mathrm{MRE}=\frac{1}{n}\sum_{i=1}^{n}\left|X_{Ci}(k)-X_{Ri}\right|$$

The actual horizontal displacements of the measuring points are taken as the benchmark, and the MAEs are minimized as the optimization objective. The particle swarm optimization (PSO) method is adopted to define the correction coefficients *k* for different deformation segments. A detailed formula description of PSO was presented in [39]. In 1995, the PSO algorithm was developed by Eberhart based on animal behaviors such as fish schooling and birds flocking, and it has the advantages of simplicity, a fast convergence speed and fewer parameters. The PSO algorithm includes two equations. The first equation involves updating the position of the particle:

(7) $$x^{i}(t+1)=x^{i}(t)+v^{i}(t+1)$$

**Figure 4:**
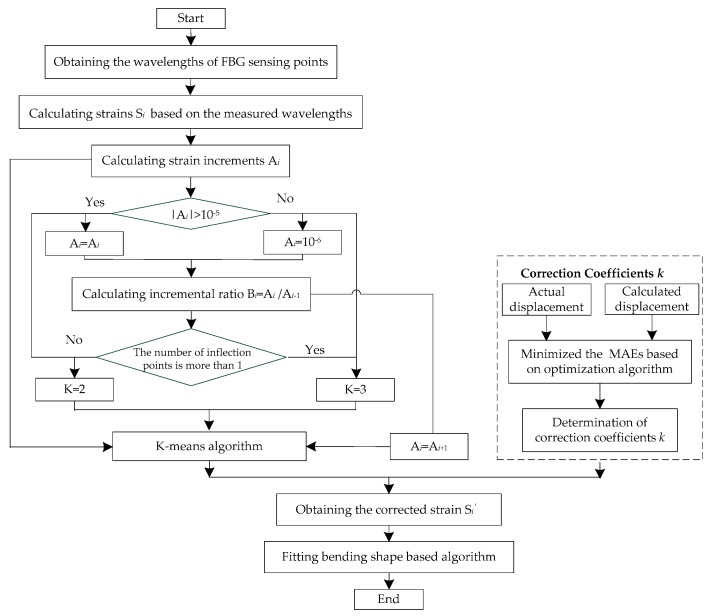
Flow chart of the segmental correction method based on strain increments at sensing points.

The second equation involves updating the velocity of a particle:

(8) $$v^{i}(t+1)=wv^{i}+C_{1}r_{1}(p_{\mathrm{best}}^{i}(t)-x^{i}(t))+C_{2}r_{2}(G_{\mathrm{best}}-x^{i}(t))$$

where *x^i^* (*t*) and *x^i^* (*t* + 1) express the position vectors of particle *i* at time *t* and *t*+1, respectively; *v* represents the velocity vector of the particle, and *w* is the inertia weight parameter that indicates the impact of the previous velocity; *C*_1_ and *C*_2_ represent the acceleration coefficients, which are called the cognition learning factor and the social learning factor, respectively; *r*_1_ and r_2_ are random numbers in the range of [0,1], and $p_{best}^{i}(t)$ and *G*_best_ represent the local best and the global best, respectively. Each particle has a velocity vector and its physical location in space. In the process of motion, the particle can remember the local best position $p_{best}^{i}(t)$ and communicate with other particles to find the global best position *G*_best_. The algorithm determines the fitness of each particle by iteration based on the objective function. The global best position can be achieved when the objective function is minimized. Therefore, the corresponding correction coefficients in different deformation segments can be obtained by using the PSO optimization algorithm. The specific flow chart was given in Figure 4.

## 3. Simulation Verification

**Figure 5:**
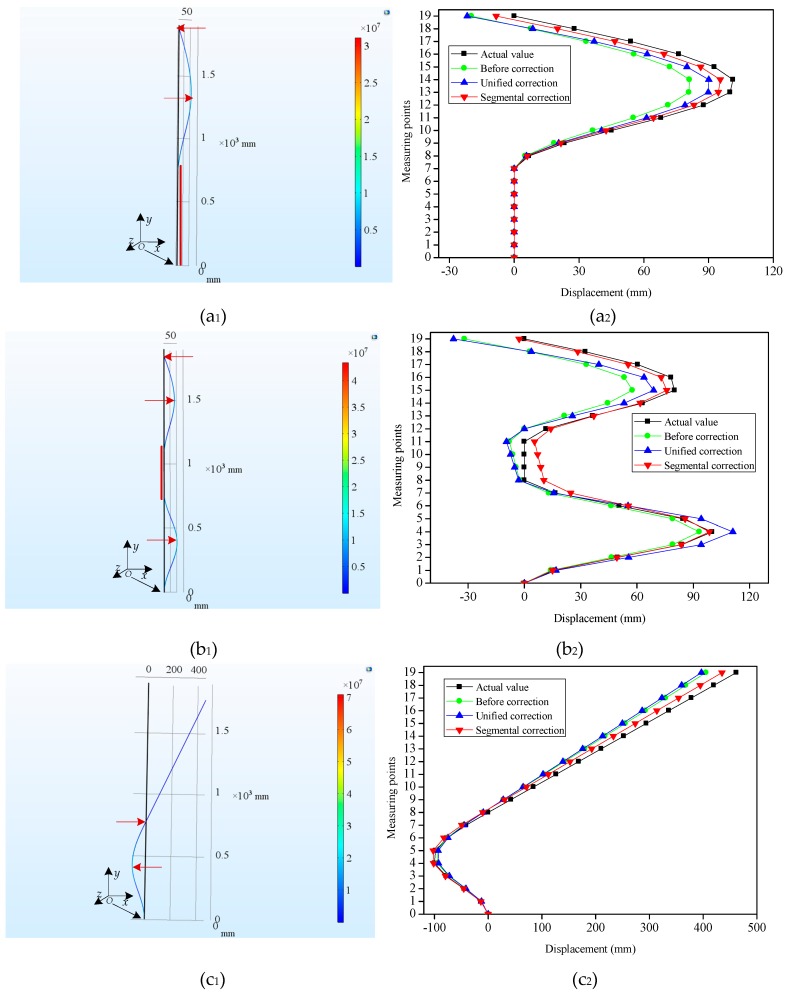
Demonstration of the simulation results of the slender plate model under different loading modes. (**a_1_**), (**b_1_**) and (**c_1_**) represented the simulation diagrams for Type 1, Type 2 and Type 3, respectively; (**a_2_**), (**b_2_**) and (**c_2_**) represented the displacements contrast of the measuring points for Type 1, Type 2 and Type 3, respectively.

In this paper, a finite element method was applied to simulate the bending shapes of the FBG flexible sensor, and the effects of displacement sensing that had been adopted in unified correction and segmental correction were compared. To remain consistent with the flexible sensor of plate-like structure used in the experiment, a slender plate model with a length of 1900 mm, a width of 10 mm and a thickness of 2 mm was established, as shown in Figure 5a_1_,b_1_,c_1_. The initial end surface (the bottom of the slender plate model) central coordinates were *O* (0, 0, 0), and the slender plate model was divided into 19 segments along the *y*-axis (length direction). Each segment was to be regarded as a beam element of the FBG flexible sensor. The slender plate material was epoxy resin, and its mechanical property parameters were listed in Table 1. In this study’s simulation, the slender plate model had a total of 18,785 meshes with minimum unit masses of 0.2418. After that, with consideration given to the geometrical nonlinearity of the material applied in the simulation, an elastic model was selected.

A displacement constraint was fixed on the bottom of the slender plate, and three different types of displacements were exerted to simulate the possible bending shapes of the flexible sensor in monitoring the internal displacements of the slope. For Type 1 (Figure 5a_1_), the displacements of the slender plate model (from the bottom to the 7-th measuring point) were constrained to zero. 100 mm and 0 mm displacements were exerted along the *x* direction at (−1, 1300, 0) and (−1, 1900, 0), respectively. For Type 2 (Figure 5b_1_), the displacements (from the 8-th measuring point to the 11-th measuring point) were constrained to zero. The displacements were imposed on the slender plate at (−1, 400, 0), (−1, 1500, 0) and (−1, 1900, 0) along the *x* direction, respectively, and the displacement values were 100 mm, 80 mm and 0 mm, respectively. For Type 3 (Figure 5c_1_), −100 mm and 0 mm displacements were exerted along the *x*-axis at (−1, 400, 0) and (−1, 800, 0), respectively.

**Figure 6:**
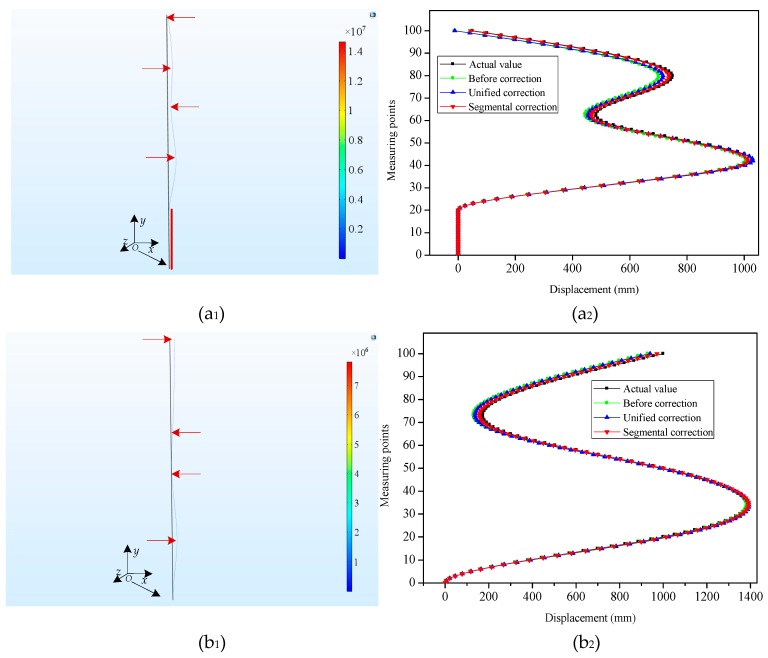

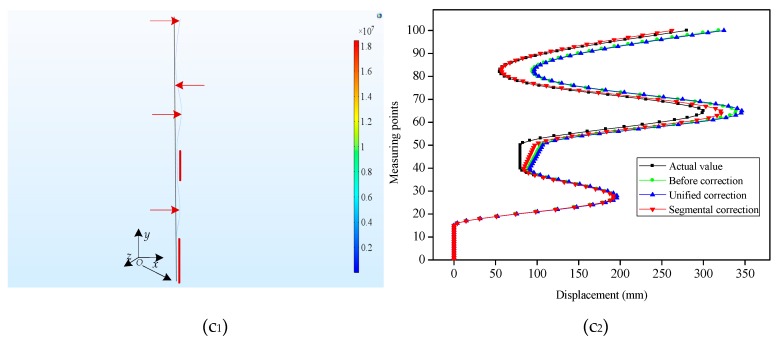
Demonstration of the simulation results of the slender plate model under different loading modes. (**a_1_**), (**b_1_**) and (**c_1_**) represented the simulation diagrams for Type 4, Type 5 and Type 6, respectively; (**a_2_**), (**b_2_**) and (**c_2_**) represented the displacements contrast of the measuring points for Type 4, Type 5 and Type 6, respectively.

Considering the practical engineering application, for the task of internal horizontal displacements monitoring in the slopes, the displacements to be measured sometimes cover a long-distance range in the vertical direction. To simulate this circumstance, a slender plate model of epoxy resin material with a length of 10,000 mm, width of 10 mm and thickness of 2 mm was also simulated. Its bending shapes were represented in Figure 6a_1_,b_1_,c_1_. For Type 4 (Figure 6a_1_), 1000 mm, 500 mm, 750 mm and 50 mm displacements were applied along the *x*-axis at (−1, 4000, 0), (−1, 6000, 0), (−1, 8000, 0) and (−1, 10,000, 0) on the slender plate model, respectively. Meanwhile, the displacements from the bottom to the 20-th measuring point were constrained and remained unchanged. For Type 5 (Figure 6b_1_), we applied 1000 mm, 100 mm, 300 mm and 1000 mm displacements along the *x* direction at (−1, 2000, 0), (−1, 5000, 0), (−1, 6500, 0) and (−1, 10,000, 0), respectively. For Type 6 (Figure 6c_1_), the displacements of two regions (from the bottom to the 15-th measuring point and from the 40-th measuring point to the 50-th measuring point) were constrained to zero and 80 mm, respectively. Meanwhile, the displacements were imposed to the slender plate model at (−1, 2500, 0), (−1, 6500, 0), (−1, 7500, 0) and (−1, 10,000, 0) along the *x* direction, respectively, and the displacement values were 180 mm, 300 mm 120 mm and 280 mm, respectively.

For the six typical bending shapes, the strain values in the coordinate points of *S_i_* (−1, 50 + 100 × *i*, 0) with *i* = 0, 1... *m* were extracted from the slender plate model. Meanwhile, the displacements in the coordinate points of *P_j_* (−1, 100 × *j*, 0) with *j* = 1, 2, ..., *n* were extracted as the standard displacement values. For Types 1, 2 and 3, *m* and *n* were 18 and 19 respectively, while for Types 4, 5 and 6, *m* and *n* were 99 and 100, respectively. By using the standard displacement values of the measuring points as the benchmark, the optimization goal was to minimize the MAEs between the reconstructed displacements and actual displacements at each measuring point. Different deformation segments under each typical bending shape of the slender plate were categorized into different classes by using the K-means clustering algorithm. Then, a PSO optimization algorithm was adopted to calculate the correction coefficients for different classes and define the unified correction coefficient of the overall deformation as a comparison at the same time. The correction coefficients for Class I and Class II were determined to be *k*_1_ = 0.96 and *k*_2_ = 1.07, respectively, and the unified correction coefficient was *k*_0_ = 1.02. To be clear, the correction coefficients (*k*_1_, *k*_2_, and *k*_0_) were the average values under three bending shapes (Type 1, 2 and 3). These correction coefficients were also employed in Types 4, 5 and 6 to contrast the displacement sensing effects under different methods.

To obtain the displacements of the measuring points under different bending shapes, the strain values which had been extracted were employed for the reconstruction of the different bending shapes of the slender plate via the shape reconstruction algorithm. Figure 5a_2_,b_2_,c_2_ and Figure 6a_2_,b_2_,c_2_ displayed the contrast between the displacements of the measuring points before and after the corrections, as well as the actual displacements under the different bending shapes. It can be seen that, having been influenced by multiple factors (such as the accumulated errors of the measuring points), there were certain errors between the initial reconstructed displacements and the actual displacements. It was observed for Types 2, 3, 4 and 5 that the absolute errors of the measuring points away from the fixed end (the bottom of the slender plate) had gradually increased (with maximum errors of 31.87 mm, 56.38 mm, 62.76 mm and 71.52 mm, respectively), and both were located at the last measuring point. However, for Types 1 and 6, the measuring points with the maximum absolute errors were located in the 10-th, and 16-th points, with absolute error values of 20.78 mm and 40.08 mm, respectively. This was determined to be due to the fact that the displacement errors of the measuring points displayed the phenomena of positive and negative error offsets. It was obvious that the initial reconstructed displacements of the measuring points had larger errors for each type.

**Figure 7:**
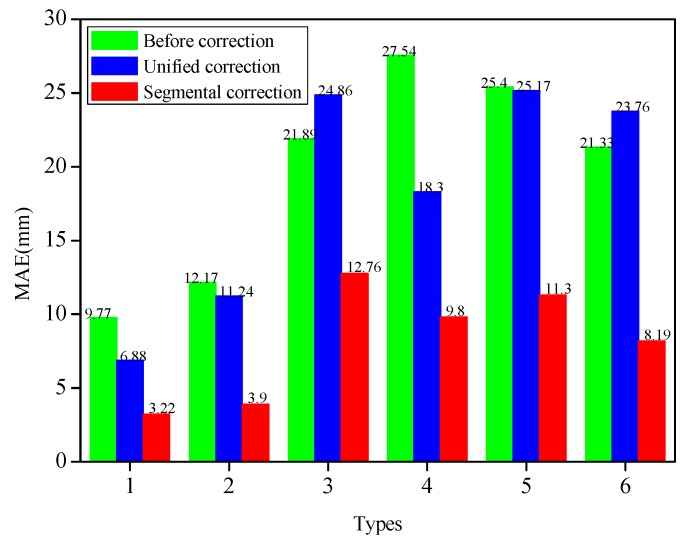
Comparison of MAEs based on different methods under six bending shapes.

Figure 7 depicted the MAEs of the measuring points under three modes of initial reconstruction, unified correction and segmental correction. The extracted strain values for each bending shape were corrected by the unified coefficient, and the MAEs of the measuring point demonstrated larger differences. For instance, for Types 1, 2, 4 and 5, the MAEs of the corrected measuring points were decreased by 2.89 mm, 0.93 mm, 9.24 mm and 0.23 mm, respectively, compared with those before the correction. Nevertheless, for Types 3 and 6, the MAEs of the measuring points were augmented by unified corrections, which were 2.97 mm and 2.43 mm, respectively. Therefore, this fully testified to the fact that it was not competent to use the unified correction method to correct different bending shapes. However, the absolute errors of the six bending shapes had been determined to decline significantly after the strain values of different deformation segments for each bending shape were corrected by the segmental coefficients. The MAEs of Types 1, 2, 3, 4, 5 and 6 after the segmental corrections were 3.22 mm, 3.90 mm, 12.76 mm, 9.8 mm, 11.3 mm and 8.19 mm, respectively. The MAEs had dwindled by 6.55 mm (Type 1), 8.27 mm (Type 2), 9.13 mm (Type 3), 17.74 mm (Type 4), 14.1 mm (Type 5), and 13.14 mm (Type 6), respectively, following the segmental corrections. The segmental correction coefficients determined by Types 1, 2, and 3 were also applied to Types 4, 5, and 6 with good correction performances. The segmental coefficients correction showed its advantages in correcting the displacement errors of the measuring points with different bending shapes when compared with the unified coefficient correction. These findings indicated that the segmental correction method had the ability to improve the displacement accuracy of the measuring points with different bending shapes, which confirmed that it was necessary and feasible to use segmental correction for the various bending shapes.

## 4. Experimental Section and Analyses

### 4.1. Design of the FBG Flexible Sensor

**Figure 8:**
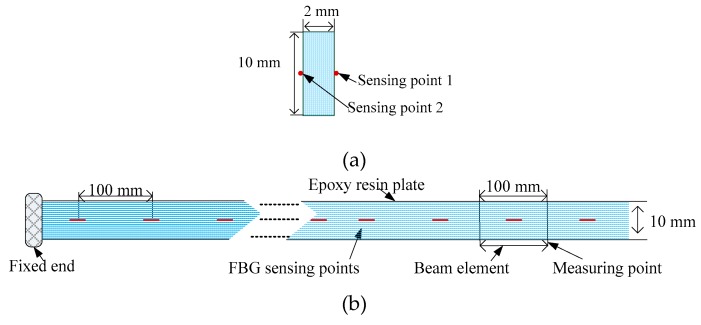
Schematic diagram of the FBG flexible sensor. (**a**) Side view of the sensor; (**b**) Front view of the sensor.

The operability and convenience of the experiment were considered, and an FBG flexible sensor with a plate-like structure was designed in the experiment. As shown in Figure 8, to fabricate the sensor, an epoxy resin plate was adopted as a flexible substrate, which had the characteristics of a high strength, corrosion resistance, and high temperature resistance. The flexible plate length was 1900 mm, the width was 10 mm and the thickness was 2 mm, which could recover the original shape after many measurements, so as to obtain a good repeatability for the displacements measurement. The FBG sensing points were symmetrically attached on the front side surface and rear side surface along the central axis using a commercial adhesive. The intervals between the adjacent FBG sensing points were 100 mm, and FBGs were manufactured using the phase mask technology by Suzhou NanZee Sensing Technology Co., Ltd., Suzhou, China. The detailed parameters were set out in Table 2. In practical engineering applications, the flexible substrate surface is grooved along the axis, and then the FBG sensors are fixed in the grooves. Besides, the outer part of the FBG flexible sensor is protected by a casing. These measures and methods maximize the protection of the sensing points and ensure the accuracy of the strain transmission.

### 4.2. Temperature Sensing Experiment

The flexible plate was divided into 19 beam elements by the FBG sensing points. When the flexible sensor was bent, the sensing point 1 and sensing point 2 (see Figure 8a) would respectively be stretched and compressed because the sensing points were symmetrically placed on both sides of the beam element. Thus, the bending-induced wavelength shifts of the two sensing points were identical in the opposite direction. Meanwhile, the temperature-induced wavelength shifts demonstrated the same direction. Therefore, the strain differences between the two sensing points on each beam element were used to eliminate the influence of the temperature. Then, the strain value could be calculated as:

(9) $$\Delta \varepsilon =\frac{1}{2}(\Delta \varepsilon_{1}-\Delta \varepsilon_{2})=\frac{1}{2(1-P_{e})}(\frac{\Delta \lambda_{1}}{\lambda_{1}}-\frac{\Delta \lambda_{2}}{\lambda_{2}})$$

**Figure 9:**
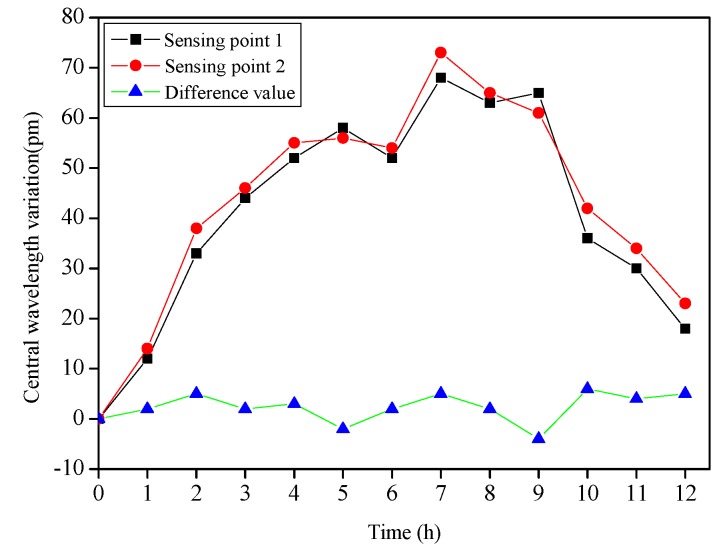
The central wavelength variations of two sensing points.

In engineering applications, the FBG flexible sensor is buried inside the slope, and the temperature around the sensor varies greatly throughout the year. Therefore, it is required for the sensor to have the ability to conduct a temperature self-compensation to avoid measurement deviations caused by temperature. A temperature sensing experiment was performed at room temperature for the fabricated FBG flexible sensor. The sensor was in a free state, and a four-channel FBG demodulation instrument SM130 (MOI) was employed to collect the central wavelength of each sensing point, whose wavelength measurements ranged from 1510 nm to 1590 nm with a demodulation precision of 1 pm. The central wavelengths of all FBG sensing points were recorded in real time from 8 a.m. to 8 p.m. The central wavelength variations of two sensing points on one of the beam elements were shown in Figure 9. The central wavelength maximum variations of the sensing point 1 and sensing point 2 were 68 pm and 73 pm, respectively. It was evident that temperature fluctuations could cause measurement errors. However, the differences of the central wavelength variations between the two sensing points were concentrated near zero, and the maximum fluctuation was only 6 pm. In addition, the maximum fluctuation values of the difference between the central wavelength variations of the two sensing points on the other beam elements were in the range of 10 pm. Therefore, it could be considered that the flexible sensor achieved an excellent performance in terms of temperature self-compensation.

### 4.3. Experimental Validations of the Proposed Correction Method

**Figure 10:**
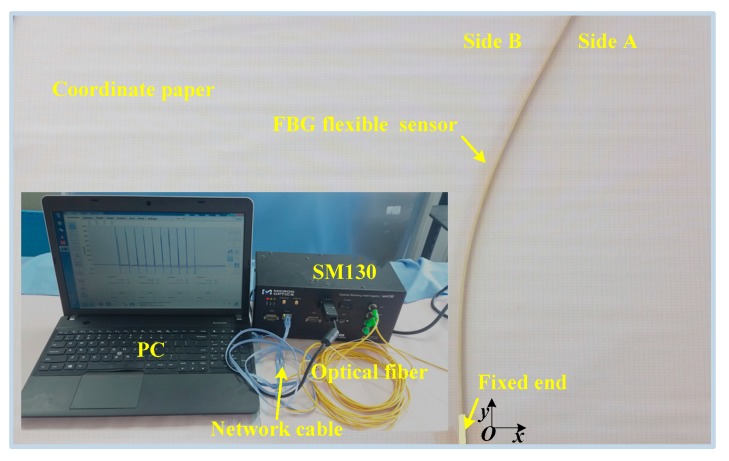
Schematic diagram of the experimental facility.

To verify the feasibility and effectiveness of the segmental correction method based on the strain increments at the sensing points, the displacements calibration experiment was implemented in this study. Figure 10 displays the experimental setup. One end of the FBG flexible sensor was fixed on the calibration platform with coordinate paper (cell: mm^2^). The coordinate of the fixed point on side A was *O* (0, 0), and the coordinates of each measuring point on side A were *P_i_* (0, 100 × *i*), with *i* = 1, 2, …, 19. The tail fiber was connected to the SM130, and the central wavelength data of the all FBG sensing points collected by the SM130 demodulation were transmitted to the computer via a network cable. Then, the displacements were exerted to the different locations of the sensor for the formation of the different bending shapes, and the displacements of each measuring point were detected by electronic digital caliper (resolution: 0.01 mm). It should be noted that the wavelengths of the sensing points on side B minussymmetrical the wavelengths of the sensing points on side A, which was adopted to achieve the purpose of eliminating the influence of temperature.

**Figure 11:**
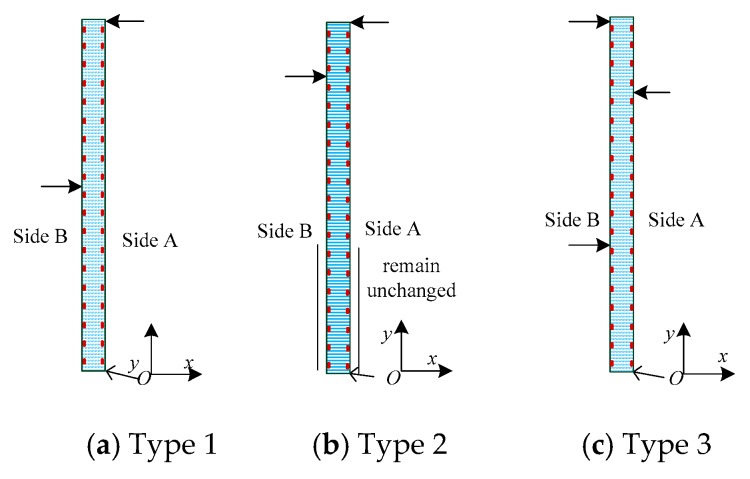
Schematic diagram of the different bending shapes of the FBG flexible sensor under different loading modes. (**a**) shows middle landslides and deep landslides; (**b**) shows mainly middle landslides and a deep horizontal displacement remained essentially unchanged; (**c**) shows shallow landslides and deep landslides.

**Figure 12:**
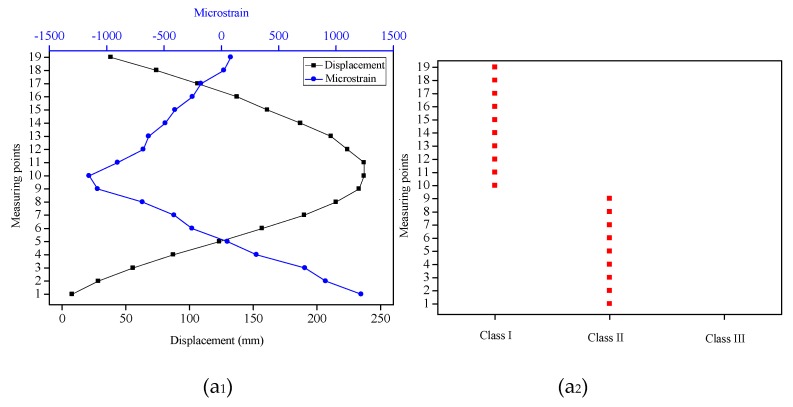

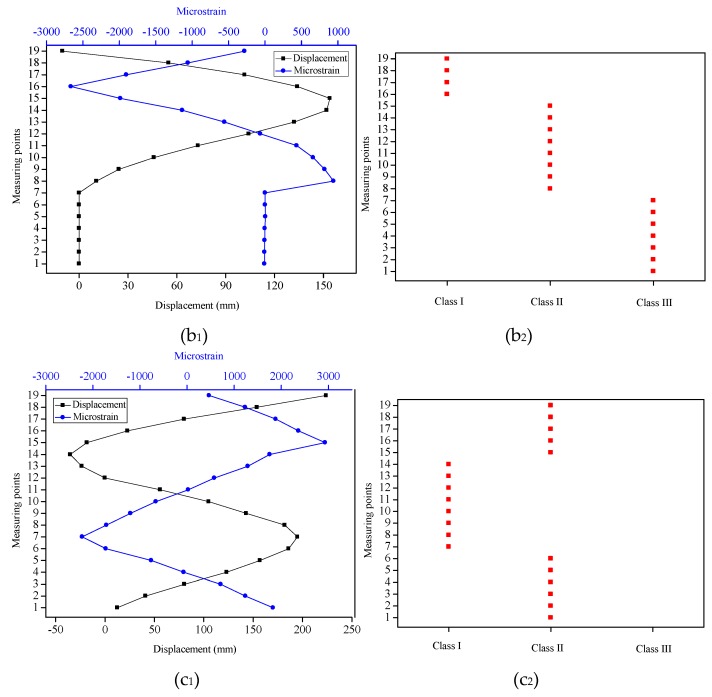
The displacements of the measuring points and micro-strains of the sensing points for three types and the clustering results. (**a_1_**), (**b_1_**) and (**c_1_**) represented the displacements and strains for Type 1, Type 2 and Type 3, respectively. (**a_2_**), (**b_2_**) and (**c_2_**) represented the clustering results for Type 1, Type 2 and Type 3, respectively.

The experiment was conducted on three typical bending shapes of the FBG flexible sensor that may occur in the engineering application, and three different sizes of displacements were applied to each type. Type 1 (Figure 11a) included middle landslides and deep landslides. To simulate the bending shapes of the flexible sensor for Type 1, three different sizes of displacements were applied to the sensor at (0, 1000) and (0, 1900) along the *x* direction, at 178.72 mm/15.34 mm, 237.2 mm/38.36 mm, and 278.06 mm/55.47 mm, respectively. Mainly middle landslides and a deep horizontal displacement remained essentially unchanged and were expressed in Type 2 (Figure 11b). Thus, the displacements were subjected along the *x*-axis at (0, 1600) and (0, 1900), at 134.18 mm/−10.05 mm, 158.61 mm/−8.14 mm, and 178.17 mm/−5.42mm, respectively, while at the same time the displacements from the measuring point 1 to measuring point 7 remained basically unchanged. Shallow landslides and deep landslides occurred in Type 3 (Figure 11c) and the middle belonged to the passive sliding zone. In the course of the experiment, the displacements were exerted on the sensor at (0, 700), (0, 1500) and (0, 1900) along the *x* direction, respectively, and the displacement values in the three cases were 162.48 mm/−8.26 mm/172.12 mm, 195.04 mm/−19.19 mm/224.12 mm, and 215.07 mm/−25.14 mm/174.49 mm, respectively. For the displacement values in the three cases for each bending shape, three repetitions were performed in each case. Then, SM130 was employed to collect the central wavelength data of each sensing point on both sides of the flexible sensor. The actual displacement values were achieved using an electronic digital caliper when the displacements were stable. A displacement case was randomly selected for each bending shape, and the average of three repetitive experimental data values was taken. The displacements of the measuring points and micro-strains of the sensing points for each type were illustrated in Figure 12a_1_,b_1_,c_1_.

For each type, we calculated the strain increments at the sensing points and extracted them as eigenvalues to be clustered. The K-means clustering algorithm was employed to cluster the strain increments so that the deformation segments with different bending shapes of the flexible sensor could be identified. For Type 1, the 10-th strain value was the inflection point, so the K value was equal to 2, and the clustering algorithm outputted two classes. It can be seen from Figure 12a_1_ that the strain values (from the first sensing point to the 9-th sensing point) were decreased gradually, so the strain increments at these sensing points were negative values and belonged to Class II; meanwhile, the strain values from the 10-th sensing point to the 19-th sensing point were increased gradually, which should belong to Class I with positive strain increments. The clustering results were consistent with the analyses, as displayed in Figure 12a_2_. For Type 2, the strain values had two inflexion points (8-th point and 16-th point), and there were points where the strain increments were basically unchanged. Thus, K was assigned to 3, and different deformation segments were clustered into three classes. Figure 12b_1_ demonstrated that the strain increments hardly changed from the first sensing point to the 7-th sensing point; the strain increments from the 8-th point to the 15-th point were negative values, while the strain increments were positive values from the 16-th point to the 19-th point. Different deformation segments were clustered correctly based on the clustering algorithm (see Figure 12b_2_). For Type 3 (see Figure 12c_1_), although the strain values had two inflection points (7-th point and 15-th point), there were no points where the strain increments were basically unchanged. Therefore, the value set for K was 2, obtaining two classes of clustering results. In Class I, the strain increments at the sensing points (from 7-th point to 14-th point) were positive, while the negative strain increments at the sensing points (from first point to 6-th point and from 15-th point and 19-th point) were Class II. It can be seen from Figure 12c_2_ that the clustering results were consistent with the analyses. The clustering results of each type indicated that different deformation segments of the flexible sensor have been correctly categorized by the clustering algorithm.

The next step was to calculate the correction coefficients of different classes. To be specific, the actual horizontal displacements of the measuring points were taken as the benchmark, and the optimization objective was to minimize the MAEs between the reconstructed displacements based on the shape reconstruction algorithm and actual displacements at each measuring point. A PSO optimization algorithm was adopted to define the correction coefficients for different classes and to calculate the unified correction coefficient of the overall deformation as a comparison at the same time. The correction coefficients for Class I and Class II were determined to be *k*_1_ = 0.85 and *k*_2_ = 1.18, respectively, and the unified correction coefficient was *k*_0_ = 1.08. It should be noted that three correction coefficients (*k*_1_, *k*_2_, and *k*_0_) were average values in the three cases for each bending shape.

**Figure 13:**
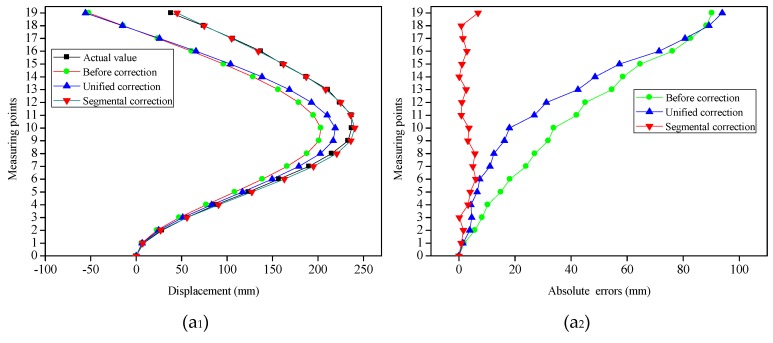

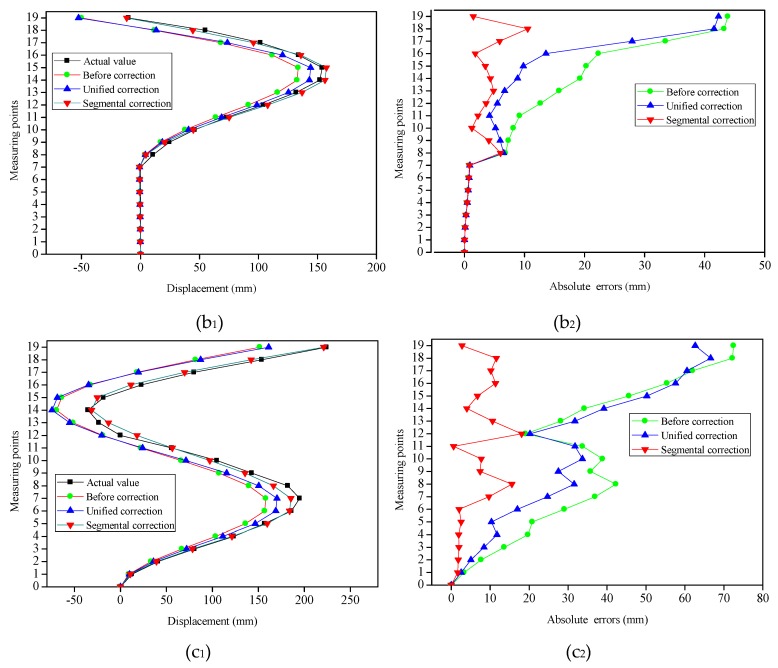
Demonstration of the experimental results under three bending shapes of the FBG flexible sensor. (**a_1_**), (**b_1_**) and (**c_1_**) represented the displacements contrast of the measuring points for Type 1, Type 2 and Type 3, respectively. (**a_2_**), (**b_2_**) and (**c_2_**) represented the absolute errors contrast of the measuring points for Type 1, Type 2 and Type 3, respectively.

Figure 13 displays the contrast between the displacements of the measuring points before and after the corrections, and the actual displacements under the three bending shapes, as well as the absolute errors of the measuring points before and after the corrections. It can be seen that there were certain errors between the initial reconstructed displacements and the actual displacements. It was observed that among these, with the increasing distances between the measuring points and fixed end, the absolute errors of Types 1 and 2 had gradually increased. The maximum absolute errors had all occurred at the 19-th measuring point (for example: 90.23 mm and 43.87 mm). The overall trend of absolute errors in Type 3 increased gradually, and the maximum error (equal to 72.47 mm) occurred at the 19-th measuring point. However, the absolute errors of some measuring points (9-th, 11-th and 12-th points) were smaller than that of the previous one. The reason for this phenomenon was that the error directions were different, and the positive and negative errors were offset. It was obvious that the initial reconstructed displacements of the measuring points had larger errors for each type.

**Figure 14:**
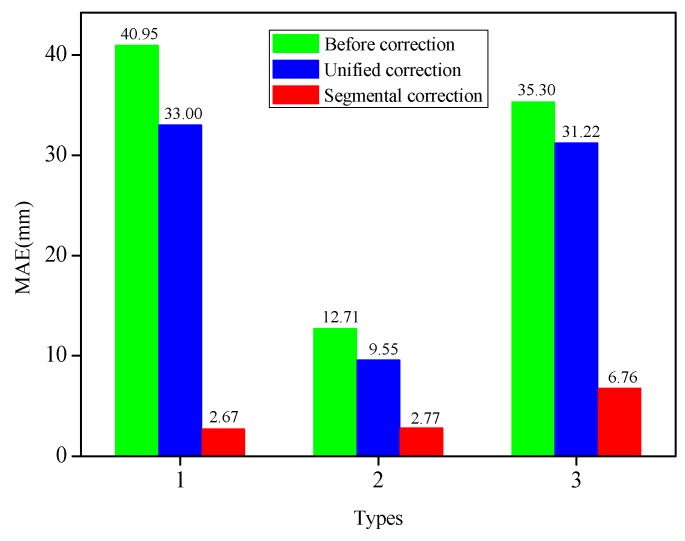
Comparison of MAEs based on different methods for Type 1, Type 2 and Type 3, respectively.

The MAEs calculated by the initial reconstruction, along with the two correction methods, were depicted in Figure 14. After the strain values of all beam elements for each bending shape were corrected by the unified coefficient, the MAEs of the measuring points displayed larger differences. For example, for Type 1, the MAE of the measuring points after the correction was at the maximum at 33.00 mm; for Type 2, the MAE was the minimum at 9.55 mm; for Type 3, the MAE was 31.22 mm. For Type 1, 2, and 3, the MAEs of the corrected measuring points were decreased by 7.95 mm, 3.16 mm, and 4.08 mm, respectively, compared with those before the correction. However, after the strain values of different deformation segments for each bending shape were corrected by the segmental coefficients, the absolute errors of the three bending shapes had been significantly reduced. The MAEs of Types 1, 2, and 3 after segmental corrections were 2.67 mm, 2.77 mm and 6.76 mm, respectively. The MAEs had been reduced by 38.28 mm (Type 1), 9.94 mm (Type 2) and 28.54 mm (Type 3), respectively, following the segmental corrections. Compared with the unified coefficient correction, the segmental coefficients correction had a better correction ability for the displacement errors of the measuring points with different bending shapes, while also improving the measurement accuracy of the FBG flexible sensor.

### 4.4. Method Scalability of the Proposed Method

**Figure 15:**
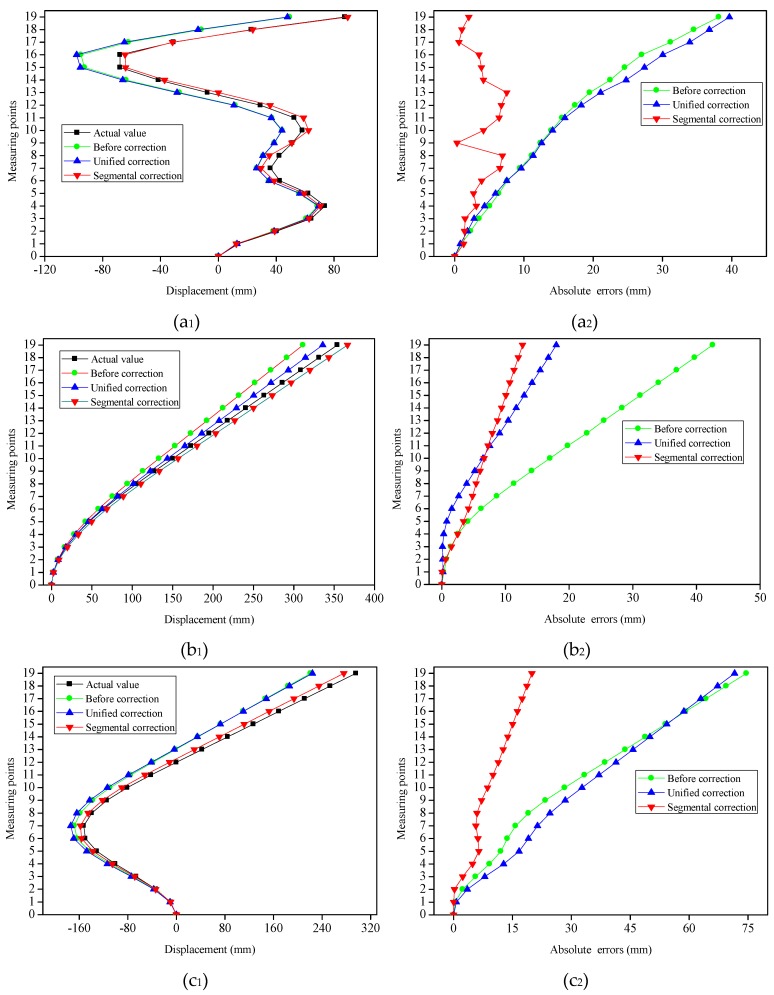
Demonstration of the experimental results under three bending shapes of the FBG flexible sensor. (**a_1_**), (**b_1_**) and (**c_1_**) represented the displacements contrast of the measuring points for Type 4, Type 5 and Type 6, respectively. (**a_2_**), (**b_2_**) and (**c_2_**) represented the absolute errors contrast of the measuring points for Type 4, Type 5 and Type 6, respectively.

It has been pointed out that the displacements at different depths of the slopes are extremely complex during the evolution process of landslides because of the double influences of natural factors and anthropogenic activities. The bending shapes of the flexible sensor have diversified characteristics in the slope measurement. To verify the scalability of the segmental correction method, three other typical bending shapes of the FBG flexible sensor have been also executed in the scalable experiment. Both the segmental correction coefficients (*k*_1_ = 0.85 and *k*_2_ = 1.18) and the unified correction coefficient (*k*_0_ = 1.08) that had been determined in the above experiment were adopted in the comparison of the displacements sensing. Type 4 (Figure 15a_1_) included middle landslides and deep landslides, and the shallow layer produced a reverse horizontal displacement influenced by rainfall. To realize the bending shapes of the flexible sensor for Type 4, 73.78 mm, 36.07 mm, 29.14 mm, −67.96 mm and 87.49 mm displacements were imposed to the flexible sensor at (0, 400), (0, 700), (0, 1200), (0, 1600) and (0, 1900) along the *x* direction, respectively. The overall slip of slope body occurred in Type 5 (Figure 15b_1_). Thus, we applied a 149.56 mm displacement along the *x*-axis at (0, 1000). For Type 6 (Figure 15c_1_), landslides occurred in the shallow layer and middle layer. Meanwhile, it engendered a reverse horizontal displacement in the deep layer due to the groundwater being affected. In order to achieve this goal, the displacements were exerted on the sensor at (0, 600) and (0, 1200) along the *x* direction, respectively, and the displacement values were −149.89 mm and 0.36 mm, respectively. Repeated experiments were performed three times for each bending shape, and the average data were taken. Figure 15 displays the contrast of the displacements sensing under four modes (actual value, initial reconstruction, unified correction and segmental correction), as well as the absolute errors of the measuring points before and after the corrections.

**Figure 16:**
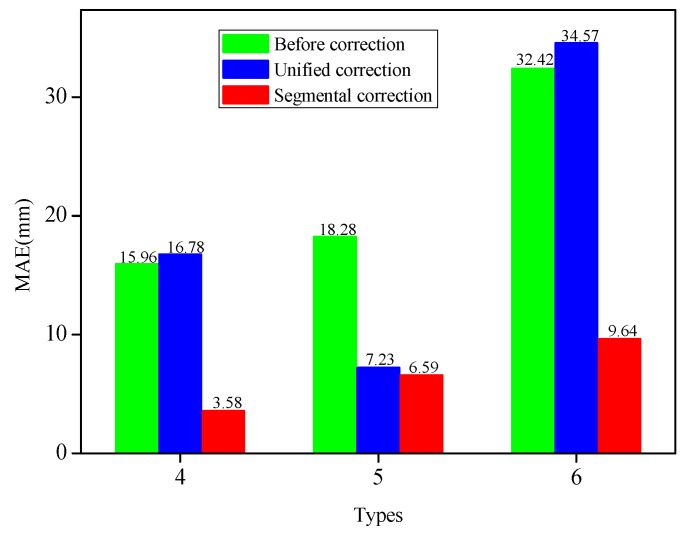
Comparison of MAEs based on different methods for Type 4, Type 5 and Type 6, respectively.

It was obvious that the initial reconstructed displacements of the measuring points had larger errors for each type (see Figure 15a_2_,b_2_,c_2_). The absolute errors had gradually increased, and the maximum absolute errors had all occurred to the 19-th measuring point (for example: 38.13 mm, 42.56 mm and 74.62 mm). The MAEs calculated by the initial reconstruction, along with the two correction methods, were depicted in Figure 16. Following the unified coefficient correction, the MAEs of corrected measuring points were decreased by 11.05 mm for Type 5. On the contrary, for Types 4 and 6, the MAEs of the measuring points were raised by unified corrections, which were 0.82 mm and 2.15 mm, respectively. Therefore, this exposed the inefficiencies of using a unified correction coefficient to correct the different bending shapes. The absolute errors of each type had been significantly reduced following the segmental coefficients. The MAEs of Types 4, 5 and 6 were 3.58 mm, 6.59 mm and 9.64 mm, respectively, following the segmental corrections. The MAEs had dwindled by 12.38 mm (Type 4), 11.69 mm (Type 5), and 22.78 mm (Type 6), respectively. Consequently, the segmental correction method and the correction coefficients determined by Types 1, 2 and 3 have also achieved favorable performances in the displacement errors correction of the measuring points in Types 4, 5 and 6. For each type, the displacement of each measuring point was *X_i_*, with *i* = 1, 2, ..., 19. The mean absolute displacement *x* of the measuring points was $\frac{1}{19}\sum_{i=1}^{19}\left|X_{i}\right|$. The MAE percentage can be calculated by MAEs/*x* × 100%. The MAE percentages of the measuring points based on different methods under six bending shapes were reflected in Table 3. The MAE percentages of Types 1, 2, 3, 4, 5 and 6 after segmental corrections were 1.87%, 5.28%, 6.98%, 7.62%, 4.16% and 8.31%, respectively. The MAE percentages had been declined by 26.83% (Type 1), 18.94% (Type 2), 29.49% (Type 3), 26.35% (Type 4), 7.39% (Type 5), and 19.65% (Type 6), respectively, following the segmental corrections. The MAE percentages of each bending shapes were observed to be relatively low.

In all of the experimental operations, the spectrum of the sensing points displayed excellent performances without distortion. In practical engineering applications, the FBG flexible sensor is mounted in the casing. With the protection of the casing, the influence of the shear strain around the sensor is eliminated, and the sensing points are protected at the same time. Even if the FBG flexible sensor undergoes various bending deformations, the strain increments at the sensor points can only be in three classes at most, which is consistent with the scheme proposed in this paper. By using the proposed correction method, we can obtain reliable data to realize accurate measurements of displacement in the slope. Compared with the dimension of the fabricated sensor, the displacement of the deformation segments meets the practical application requirements. Therefore, early warning is provided to the public before the plastic deformation of the FBG flexible sensor occurs. These findings indicated that the proposed segmental correction method was feasible, and the method was found to be effective in improving the displacement field sensing accuracy of the FBG flexible sensor in slope-related applications.

## 5. Conclusions

In this research, a self-correction displacement profiles sensing method based on strain increment clustering was proposed, which had ability to improve the accuracy of the FBG flexible sensor for measuring the horizontal displacement profiles inside the slope. Unlike the traditional correction method which calibrated the entire deformed shape and then applied the unified coefficient to correct the structures to be measured, our method was competent in automatically identifying different deformation segments using a K-means clustering algorithm based on strain increments at sensing points. After that, the correction coefficients of the deformation segments were determined by calibrating the typical bending shapes of the FBG flexible sensor in the measurement of the slope displacement profiles, and the PSO optimization algorithm was employed to achieve their optimal values. In addition, a finite element method was adopted to simulate six typical bending shapes of a slender plate model, and the necessity and feasibility of the proposed correction method were analyzed in detail. Furthermore, the operability and convenience of the experiment needed to be taken into consideration, and an FBG flexible sensor was fabricated by integrating the FBG sensing points with a flexible epoxy resin plate substrate. A temperature sensing experiment was performed to determine that the influences of temperature fluctuations could be eliminated by using differences of the central wavelength variations between two sensing points in each beam element. Finally, in order to validate the feasibility and effectiveness of the proposed method, the displacement calibration experiment was implemented. The clustering results of each type indicated that different deformation segments of the flexible sensor have been correctly categorized by the K-means clustering algorithm. Following the segmental corrections, the MAE percentages of six types were 1.87% (Type 1), 5.28% (Type 2), 6.98% (Type 3), 7.62% (Type 4), 4.16% (Type 5) and 8.31% (Type 6), respectively, which was an improvement of the accuracy by 26.83%, 18.94%, 29.49%, 26.35%, 7.39%, and 19.65%, respectively. The contrast of displacement sensing in four modes (actual value, initial reconstruction, unified correction and segmental correction) fully illustrated the feasibility and validity of the segmental correction method. Overall, these findings indicated that a self-correction displacement profiles sensing method based on strain increment clustering was empowered to effectively improve the displacement accuracy of measuring points with different bending shapes for the FBG flexible sensor. This method has broad application prospects in the measurement of displacement fields inside a slope.

The above operations were carried out in the laboratory, and excellent results were obtained. Considering the practicability and scalability of the proposed method, the next step is to apply the manufactured sensors to practical engineering and to verify their measurement performance. Full consideration is given to the protection of FBG sensing points and the plastic deformation of sensors under a large displacement deformation. In the future, more work should be investigated to promote the performance of the FBG flexible sensor.

## Figures and Tables

**Table 1:** Mechanical property parameters of epoxy resin materials.

Parameters	Values
elasticity modulus	24 GPa
shearing modulus	8.7 GPa
mass density	1950 Kg/m^3^
tensile strength	300 MPa
Poisson ratio	0.38

**Table 2:** The detailed parameters of the fiber Bragg grating (FBG) sensing points.

Parameters	Description
fiber type	single mode fiber
grating length	10 mm
bandwidth 3 dB	<0.2 nm
side lobe suppression ratio	>15 dB

**Table 3:** The MAE percentages based on different methods under six bending shapes.

Bending Shapes	Type 1	Type 2	Type 3	Type 4	Type 5	Type 6
Before Correction	28.70%	24.22%	36.47%	33.97%	11.55%	27.96%
Unified Correction	23.13%	18.20%	32.26%	35.72%	4.57%	29.81%
Segmental Correction	1.87%	5.28%	6.98%	7.62%	4.16%	8.31%
